# Tuning Scorpion Toxin Selectivity: Switching From K_V_1.1 to K_V_1.3

**DOI:** 10.3389/fphar.2020.01010

**Published:** 2020-07-07

**Authors:** Andrei M. Gigolaev, Alexey I. Kuzmenkov, Steve Peigneur, Valentin M. Tabakmakher, Ernesto L. Pinheiro-Junior, Anton O. Chugunov, Roman G. Efremov, Jan Tytgat, Alexander A. Vassilevski

**Affiliations:** ^1^Shemyakin-Ovchinnikov Institute of Bioorganic Chemistry, Russian Academy of Sciences, Moscow, Russia; ^2^Toxicology and Pharmacology, KU Leuven, Leuven, Belgium; ^3^School of Biomedicine, Far Eastern Federal University, Vladivostok, Russia; ^4^Department of Applied Mathematics, National Research University Higher School of Economics, Moscow, Russia; ^5^Moscow Institute of Physics and Technology (State University), Dolgoprudny, Russia

**Keywords:** scorpion venom, neurotoxin, voltage-gated potassium channel, potassium channel blocker, molecular modeling, molecular dynamics

## Abstract

Voltage-gated potassium channels (K_V_s) perform vital physiological functions and are targets in different disorders ranging from ataxia and arrhythmia to autoimmune diseases. An important issue is the search for and production of selective ligands of these channels. Peptide toxins found in scorpion venom named KTx excel in both potency and selectivity with respect to some potassium channel isoforms, which may present only minute differences in their structure. Despite several decades of research the molecular determinants of KTx selectivity are still poorly understood. Here we analyze MeKTx13-3 (Kalium ID: α-KTx 3.19) from the lesser Asian scorpion *Mesobuthus eupeus*, a high-affinity K_V_1.1 blocker (IC_50_ ~2 nM); it also affects K_V_1.2 (IC_50_ ~100 nM), 1.3 (~10 nM) and 1.6 (~60 nM). By constructing computer models of its complex with K_V_1.1–1.3 channels we identify specific contacts between the toxin and the three isoforms. We then perform mutagenesis to disturb the identified contacts with K_V_1.1 and 1.2 and produce recombinant MeKTx13-3_AAAR, which differs by four amino acid residues from the parent toxin. As predicted by the modeling, this derivative shows decreased activity on K_V_1.1 (IC_50_ ~550 nM) and 1.2 (~200 nM). It also has diminished activity on K_V_1.6 (~1500 nM) but preserves K_V_1.3 affinity as measured using the voltage-clamp technique on mammalian channels expressed in *Xenopus* oocytes. In effect, we convert a selective K_V_1.1 ligand into a new specific K_V_1.3 ligand. MeKTx13-3 and its derivatives are attractive tools to study the structure-function relationship in potassium channel blockers.

## Introduction

It is believed that potassium (K^+^) channels arose near the time of life origin on the earth. K^+^ channels are key membrane proteins of all living organisms, and about 80 genes encoding the main α-subunits are found in mammalian genomes (Alexander et al., 2019). The most prevalent family of K^+^ channels in mammals is voltage-gated potassium channels (K_V_s) that includes 40 isoforms (Attali et al., 2019). These proteins control neuronal excitability, heart rate, muscle contraction, hormonal secretion, cell proliferation, etc. It is not surprising that modulation of K_V_s provokes changes in the physiology of a cell or even of the whole organism (Hille, 2001).

K_V_1.3 is one of the most studied and pharmacologically important isoforms of K^+^ channels. At least two major directions of biomedical research are associated with this type of channel. First, K_V_1.3 in T lymphocytes is a validated target for diverse autoimmune diseases, such as multiple sclerosis, rheumatoid arthritis, and type 1 diabetes (Chandy et al., 2004; Beeton et al., 2006; Feske et al., 2012). Second, this protein is a crucial participant in a number of cancers because it is necessary for cell proliferation, malignant angiogenesis, and metastasis (Pardo and Stühmer, 2014; Chandy and Norton, 2016; Teisseyre et al., 2019). For both of these directions selective and effective inhibitors of K_V_1.3 are desirable. Novel ligands and their derivatives are considered as promising molecular instruments in K_V_1.3 research and are exploited as templates in drug design (Wulff and Zhorov, 2008; Chandy and Norton, 2017; Prosdocimi et al., 2019). Active compounds affecting K_V_1.3 can be obtained from different natural sources, such as plant extracts and animal venoms (King, 2011; Norton and Chandy, 2017), as well as synthesized *de novo* (Schmitz et al., 2005; Hendrickx et al., 2020).

Scorpion venom serves as an abundant source of toxins acting as K^+^ channel ligands (KTx), which have evolved and been selected for a highly efficient interaction with their molecular targets, including K_V_s (Kuzmenkov et al., 2015a). According to Kalium database (https://kaliumdb.org/), these compounds are polypeptides containing 23 to 78 amino acid residues and cross-linked by two to four intramolecular disulfide bonds (Kuzmenkov et al., 2016a; Tabakmakher et al., 2019). A dominating number of known KTx adopts the CSα/β (cysteine-stabilized α-helix and β-sheet) fold, but some of them present other types of fold (Mouhat et al., 2004; Kuzmenkov et al., 2015a). A number of structural and pharmacological findings pinpointed important determinants in the interfaces of K_V_s and KTx contact that contribute to toxin selectivity (Aiyar et al., 1995; Hidalgo and MacKinnon, 1995; Gross and MacKinnon, 1996; Giangiacomo et al., 2004). Perhaps most importantly, obtaining the crystal structure of the K_V_1.2/K_V_2.1 paddle chimera in complex with charybdotoxin (ChTx) (Banerjee et al., 2013) highlighted the key amino acids involved in the interaction and opened new opportunities for scaffold engineering of more selective KTx (Han et al., 2008; Kuzmenkov et al., 2018).

Earlier, we identified and purified MeKTx13-3 toxin (Kalium ID: a-KTx 3.19, UniProt ID: C0HJQ6, 37 residues, three disulfide bonds) from the venom of the lesser Asian scorpion *Mesobuthus eupeus* (Kuzmenkov et al., 2015b). We performed pharmacological profiling of this KTx on several isoforms of K_V_s and found that it is active on K_V_1.1–1.3 and 1.6 with half-maximal inhibitory concentration (IC_50_) values of ~2, 100, 10, and 60 nM, respectively. The toxin preferably blocked K_V_1.1, however, cross-reactivity with K_V_1.3 was also observed (Kuzmenkov et al., 2019). Since a large number of KTx inhibits both K_V_1.1 and 1.3 in a similar manner (Mouhat et al., 2005; Takacs et al., 2009; Gao et al., 2010), the goal of our present work is to identify molecular determinants responsible for the interaction with different channel isoforms and switch the selectivity of MeKTx13-3 from K_V_1.1 to K_V_1.3.

## Materials and Methods

### Ethics Statement

This study strictly complied with the World Health Organization’s International Guiding Principles for Biomedical Research Involving Animals. The research was carried out in AAALAC accredited organization according to the standards of the Guide for Care and Use of Laboratory Animals (8th edition, Institute for Laboratory Research of Animals). All experiments were approved by the Institutional Policy on the Use of Laboratory Animals of the Shemyakin-Ovchinnikov Institute of Bioorganic Chemistry Russian Academy of Sciences (Protocol Number 267/2018; date of approval: 28 February 2019).

### Homology Modeling of Toxins and Their Complexes With K_V_s

Since the amino acid sequence of MeKTx13-3 is identical to that of BmKTX (Romi-Lebrun et al., 1997), the known 3D structure of the latter (PDB ID: 1BKT) (Renisio et al., 2000) was used in our work. K_V_1.1 model was generated in MODELLER 9.19 (Webb and Sali, 2016) using the K_V_1.2 structure (PDB ID: 3LUT) (Chen et al., 2010) as a template. K_V_1.3 model has been generated previously (Kuzmenkov et al., 2017; Kuzmenkov et al., 2018; Berkut et al., 2019) using an analogous procedure.

Complexes of MeKTx13-3 with K_V_s were modeled considering that the toxin interacts with the channels similarly to ChTx, one of the most thoroughly studied KTx (Goldstein et al., 1994). The model of the complex of MeKTx13-3 with K_V_1.2 was built on the basis of the K_V_1.2/2.1–ChTx complex crystal structure (Banerjee et al., 2013): the structure of MeKTx13-3 was spatially aligned with the structure of channel-bound ChTx, which was subsequently replaced by the aligned toxin. Complexes with K_V_1.1 and 1.3 were generated similarly, but the first step was spatial alignment of the channel models with the K_V_1.2/2.1 chimera (Kuzmenkov et al., 2017; Kuzmenkov et al., 2018; Berkut et al., 2019).

### Molecular Dynamics Simulations

The resulting complexes of MeKTx13-3 with K_V_s were placed inside a lipid bilayer mimicking a neuronal membrane. We used a pre-equilibrated fragment of bilayer (7.0 × 7.0 × 13.5 nm^3^; 1-palmitoyl-2-oleoyl-sn-glycero-3-phosphocholine/1-palmitoyl-2-oleoyl-sn-glycero-3-phosphoethanolamine/cholesterol, POPC : POPE : Chl; 100:50:50 molecules, respectively, solvated with 14172 water molecules) that has been described in detail in our previous works (Berkut et al., 2019); some phospholipid and Chl molecules were removed to provide room for the protein. The TIP3P water model (Jorgensen et al., 1983) and the required number of Na^+^ ions (to maintain electroneutrality) were used for resolvation. All systems were equilibrated (heated up to 37°C) during 100 ps of molecular dynamics (MD) simulation. Positions of the channel C^α^ atoms of residues not involved in the channel pore vestibule, as well as the N^ϵ^ atom of Lys26 in MeKTx13-3 were restrained during the equilibration to prevent destabilization of the initial complex. Systems were then subjected to 500 ns of MD. All simulations were performed with the GROMACS software (Abraham et al., 2015) (version 2018) using the AMBER99SB-ILDN parameters set (Klepeis et al., 2010). Simulations were carried out with a time step of 2 fs, imposing 3D periodic boundary conditions, in the isothermal-isobaric (NPT) ensemble with a semi-isotropic pressure of 1 bar using the Berendsen pressure coupling algorithm (Berendsen et al., 1984), and at a constant temperature of 37°C using the V-rescale thermostat (Bussi et al., 2007). Van der Waals interactions were truncated using a 1.5-nm spherical cut-off function. Electrostatic interactions were treated with the PME algorithm. During the simulation, the position of the N^ϵ^ atom of Lys26 in each complex was restrained inside the channel pore.

### Determination of Interaction Energy and Intermolecular Contacts

We determined the intermolecular contacts during MD and estimated residual contributions to intermolecular interaction energy based on MD trajectory using our in-house software package IMPULSE (Krylov et al., in preparation) analogously to the procedures described in our previous study (Berkut et al., 2019). Briefly, H-bonds were assigned using the parameters set from the hbond utility of GROMACS software (Abraham et al., 2015) (the distance D—A ≤ 0.35 nm and the angle D—H—A ≥ 150° for the hydrogen bond D—H···A, where D and A are the hydrogen bond donor and acceptor, respectively); salt bridges, cation-π, stacking, and hydrophobic contacts were calculated using algorithms described in our previous works (Pyrkov and Efremov, 2007; Pyrkov et al., 2009). The AMBER99SB-ILDN parameters set (Klepeis et al., 2010) and 1.5 nm cutoff distance for Lennard-Jones and electrostatic interactions were used during the intermolecular short-range non-bonded interaction energy estimation, the latter being the sum of the Lennard-Jones and electrostatic terms. All drawings of 3D structures were prepared with the PyMOL Molecular Graphics System, version 1.8 (Schrödinger, LLC). Graphical representation of interaction energy profiles was performed using Python built-in libraries and the NumPy package.

### Toxin Isolation From Scorpion Venom

Natural MeKTx13-3 (α-KTx 1.19) was isolated from the same stock of *M. eupeus* venom and following the same procedure as described previously (Kuzmenkov et al., 2015b; Kuzmenkov et al., 2019).

### Recombinant Peptide Production

Recombinant MeKTx13-3 and its derivative were produced using an approach elaborated previously (Pluzhnikov et al., 2007). Briefly, the peptides were produced in a bacterial expression system as fusions with the carrier protein thioredoxin (Trx) (McCoy and LaVallie, 1994) and recombinant human enteropeptidase light chain (Gasparian et al., 2011) was used to cleave the fusions.

DNA sequences encoding MeKTx13-3 and its derivative were constructed from synthetic oligonucleotides by PCR in two steps (see **Supplemental Figure S1**). On the first step the target PCR fragments were amplified in 5 cycles using two forward primers and two reverse primers (F1, F2, R1, and R2). The four primers altogether constitute a full gene sequence. For the second step, PCR mixtures from the first step were diluted 100 times, and 1 µl of the dilution was used as a matrix; only the terminal primers (F1 and R1) were used for the amplification (**Supplemental Table S1**). The resulting PCR fragments were cloned into the expression vector pET-32b (Novagen) using *Kpn*I and *Bam*HI restriction enzymes to produce the vectors coding for the target polypeptides.

*Escherichia coli* SHuffle T7 Express cells (New England Biolabs) were transformed using the corresponding expression vectors and cultured at 30°C in LB medium to the mid-log phase. Expression was then induced by 0.2 mM Isopropyl β-d-1-thiogalactopyranoside. Cells were cultured at room temperature (24°C) overnight (16 h) and harvested by centrifugation. The cell pellet was resuspended in 300 mM NaCl, 50 mM Tris-HCl buffer (pH 8.0) and ultrasonicated. The lysate was applied to a HisPur Cobalt Resin (ThermoFisher Scientific); and the Trx-fusion proteins were purified according to the manufacturer’s protocol.

Fusion proteins were dissolved in 50 mM Tris-HCl (pH 8.0) to a concentration of 1 mg/ml. Protein cleavage with human enteropeptidase light chain (1 U of enzyme per 1 mg of substrate) was performed overnight (16 h) at 37°C. Recombinant peptides were purified by reversed-phase HPLC on a Jupiter C_5_ column (4.6 × 250 mm; Phenomenex) in a linear gradient of acetonitrile concentration (0–60% in 60 min) in the presence of 0.1% trifluoroacetic acid. The purity of the target peptides was checked by MALDI MS and analytical chromatography on a Vydac C_18_ column (4.6 × 250 mm; Separations Group) in the same acetonitrile gradient.

### Mass Spectrometry

Molecular mass measurements for natural and recombinant peptides were performed using MALDI on an Ultraflex TOF-TOF (Bruker Daltonik) spectrometer as described earlier (Kuzmenkov et al., 2016b). 2,5-Dihydroxybenzoic acid (Sigma-Aldrich) was used as a matrix. Measurements were carried out in both linear and reflector modes. Mass spectra were analyzed with the Data Analysis 4.3 and Data Analysis Viewer 4.3 software (Bruker).

### Ion Channel Expression in *Xenopus* Oocytes

All procedures were performed in agreement with the guidelines of ARRIVE (Animal Research: Reporting of In Vivo Experiments) and the “European convention for the protection of vertebrate animals used for experimental and other scientific purposes” (Strasbourg, 18.III.1986).

The major pipeline of ion channel expression in oocytes was described previously (Peigneur et al., 2011). Briefly, for the expression of K_V_ genes (rat (r)K_V_1.1, rK_V_1.2, human (h)K_V_1.3, rK_V_1.4, rK_V_1.5, and rK_V_1.6) in *Xenopus laevis* oocytes, linearized plasmids containing the respective gene sequences were transcribed using the T7 mMESSAGE-mMACHINE transcription kit (Ambion). 50 nl of cRNA solution (1 ng/nl) were injected into oocytes using a micro-injector (Drummond Scientific). The oocytes were incubated in ND96 solution: 96 mM NaCl, 2 mM KCl, 1.8 mM CaCl_2_, 2 mM MgCl_2_ and 5 mM HEPES, pH 7.4, supplemented with 50 mg/l gentamycin sulfate.

### Electrophysiological Recordings

Two-electrode voltage-clamp recordings were performed at room temperature (18–22°C) using a Geneclamp 500 amplifier (Molecular Devices) controlled by a pClamp data acquisition system (Axon Instruments) as described (Peigneur et al., 2011). Bath solution composition was ND96. K_V_ currents were evoked by 250-ms depolarization to 0 mV from a holding potential of −90 mV, followed by 250-ms pulses to −50 mV. For current–voltage relationship studies, currents were evoked by 10-mV depolarization steps. Concentration–response curves were constructed, in which the percentage of current inhibition was plotted as a function of toxin concentration. Data were fitted with the Hill equation: y = 100/[1 + (IC_50_/[toxin])^h^], where y is the amplitude of the toxin-induced effect, [toxin] is toxin concentration, and h is the Hill coefficient. Comparison of two sample means was performed using a paired Student’s t-test (p-value of 0.05 was used as a threshold of significance). All data were obtained in at least three independent experiments (n ≥ 3) and are presented as mean ± standard error of the mean.

## Results

### Computational Study

**Figure 1:**
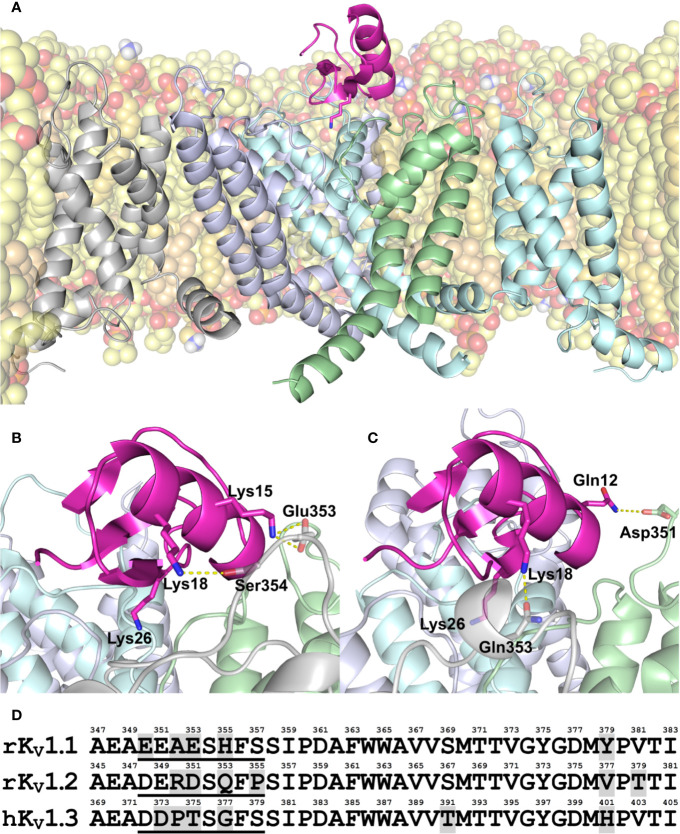
**(A–C)** Modeled structure of MeKTx13-3 in complex with K_V_1.1–1.3. **(A)** Overall structure of the K_V_1.3–MeKTx13-3 complex after 100-ns MD simulation inside a hydrated lipid bilayer membrane. Four channel α-subunits with identical sequences are color-coded. The pore domain helices of the channel subunit in the foreground and voltage-sensing domain (VSD) of the adjacent subunit, as well as extended extracellular loops of the VSDs are omitted for clarity. Lipids are shown in a semi-transparent space-filling representation; atoms are colored: oxygen, *red*; phosphorus, *orange*; nitrogen, *blue*; hydrogen of amino group, *white*; carbon of POPC, *light-yellow*; carbon of POPE, *yellow*; and carbon of cholesterol, *beige*. Some lipids are omitted for clarity. MeKTx13-3 is presented in pink; residue Lys26 (plugs the channel pore) is shown as sticks. **(B, C)** Close-up view on the channel pore vestibule area in complexes K_V_1.1–MeKTx13-3 and K_V_1.2–MeKTx13-3, respectively. Channels are shown in a semi-transparent representation. Lys26 and residues involved in the intermolecular contacts not present in the K_V_1.3–MeKTx13-3 complex are shown as sticks. Hydrogen bonds and salt bridges are shown as dashed yellow lines. Lipids are omitted for clarity. **(D)** Amino acid sequence alignment of the extracellular pore region of K_V_1.1–1.3 channels. Residue numbering is above each sequence; different residues are shaded gray; sequences of S5-P loops containing channel-specific residues are underlined.

Amino acid sequence of MeKTx13-3 is identical to BmKTX that was isolated from *Mesobuthus martensii*, a close relative of *M. eupeus* (Romi-Lebrun et al., 1997). Since the 3D structure of BmKTX is known (PDB ID: 1BKT) (Renisio et al., 2000), we used it to generate models of MeKTx13-3 in complex with K_V_1.1–1.3. The models were then subjected to MD simulations (**Figures 1A–C**).

To shed light on the molecular differences in MeKTx13-3 interaction with K_V_ isoforms, we analyzed intermolecular contacts and residual contributions to interaction energy during the MD simulations using our in-house software package IMPULSE (Krylov et al., in preparation). It was observed that in complex with K_V_1.2 the toxin does not form any cation-π or stacking contacts, unlike in complex with K_V_1.1 or 1.3 (see **Supplemental Table S2**). This observation is in good agreement with electrophysiological measurements that displayed the preferred activity of MeKTx13-3 against K_V_1.1 and 1.3 (Kuzmenkov et al., 2019). We noted the following specific contacts between the toxin and particular channel isoforms.

(1) MeKTx13-3 residue Lys15 forms an H-bond and a salt bridge with K_V_1.1 residue Glu353 (**Figure 1B**). Analogous contact with Asp351 (**Figure 1D**) is not observed in complex with K_V_1.2, apparently due to the fact that the neighboring residue Arg350 repulses Lys15 and prevents this contact formation. No contact with Thr375 was found in complex with K_V_1.3 either, presumably due to the small size of the threonine side chain and the lack of electrostatic attraction to reach Lys15.

(2) MeKTx13-3 residue Lys18 forms an H-bond with K_V_1.1 residue Ser354 and a cation-π contact with His355; in complex with K_V_1.2 it forms an H-bond with Gln353 (**Figures 1B, C**). In the complex of MeKTx13-3 with K_V_1.3 the conformation of the loop containing channel-specific residues Thr375 and Gly377 (**Figure 1D**) during MD is such that it does not reach Lys18, so no specific contacts are observed.

(3) MeKTx13-3 residue Gln12 forms an H-bond with K_V_1.2 residue Asp351 (**Figure 1C**). Analogous contact with Thr375 (**Figure 1D**) is not observed in complex with K_V_1.3 because the short threonine side chain does not reach Gln12. No contact with Glu353 is found in complex with K_V_1.1 because the conformation of the loop containing channel-specific residues during MD prevents reaching Gln12.

In addition to the observed channel-specific contacts, MeKTx13-3 residue Asp33 makes a significant positive contribution to the binding energy (negatively affects the affinity) (see **Supplemental Figure S2**) due to electrostatic repulsion with a conserved negatively charged residue in the channel vestibule (Asp377/375/399 in K_V_1.1/1.2/1.3, see **Figure 1D**).

**Figure 2:**
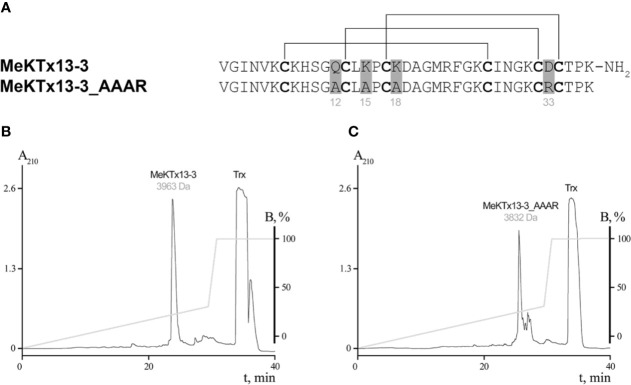
Production of MeKTx13-3 and its derivative. **(A)** Amino acid sequence alignment of MeKTx13-3 and MeKTx13-3_AAAR. Gray shading indicates the positions where replacements were introduced. Cysteine residues are in bold, and lines above the sequences indicate disulfide bonds. Take a note that recombinant analogue of MeKTx13-3 does not bear the C-terminal amidation of the natural toxin. **(B, C)** Reversed-phase HPLC separation of recombinant MeKTx13-3 and MeKTx13-3_AAAR after digestion by enteropeptidase. For target peptides the measured molecular masses are indicated.

Pharmacological profiling and detailed complex structure analysis allow us to propose several point substitutions in MeKTx13-3 for switching toxin selectivity. Since Gln12, Lys15, and Lys18 form hydrogen bonds, salt bridges, and cation-π interactions in complexes with K_V_1.1 and 1.2, the general idea of the modifications was to abolish the formation of these polar contacts. Moreover, since these toxin residues do not form specific contacts with K_V_1.3, it is reasonable to assume that such modifications will not affect the affinity to this channel isoform significantly. Therefore, we suggested a derivative of MeKTx13-3 in which Gln12, Lys15, and Lys18 are replaced by Ala to reduce toxin affinity to K_V_1.1 and 1.2, and Asp33 is replaced by Arg to increase its affinity to K_V_s (**Figure 2A**).

### Recombinant Toxin Production

The natural toxin was purified from crude venom as we described previously (Kuzmenkov et al., 2015b; Kuzmenkov et al., 2019). Recombinant MeKTx13-3 and its derivative MeKTx13-3_AAAR (MeKTx13-3 with the following replacements: Gln12Ala, Lys15Ala, Lys18Ala, and Asp33Arg) were obtained according to our common protocol (Pluzhnikov et al., 2007) using *E. coli* SHuffle B strain as an expression system. Synthetic genes encoding the peptides were cloned into the pET-32b expression vector, and Trx was used as a fusion partner to ensure a high yield of the disulfide-containing peptides with native conformation. The target peptides were produced as a result of fusion protein cleavage by enteropeptidase followed by separation using reversed-phase HPLC and identification by MALDI MS (**Figures 2B, C**). The measured molecular masses of the purified peptides were equal to the calculated values. The final yield of the peptides was ~5 mg per 1 l of bacterial culture. Note that the natural toxin is C-terminally amidated (Kuzmenkov et al., 2015b), whereas this modification is missing from the recombinant peptides.

### Electrophysiology

We first compared the activity of the natural and recombinant MeKTx13-3 (**Supplemental Figure S3** and **Supplemental Table S3**) on K_V_1.1. Recombinant peptide was less active than the native toxin (IC_50_ values of 6.7 ± 2.7 and 1.9 ± 0.2 nM, respectively), which is probably due to the lack of C-terminal amidation in the former.

**Figure 3:**
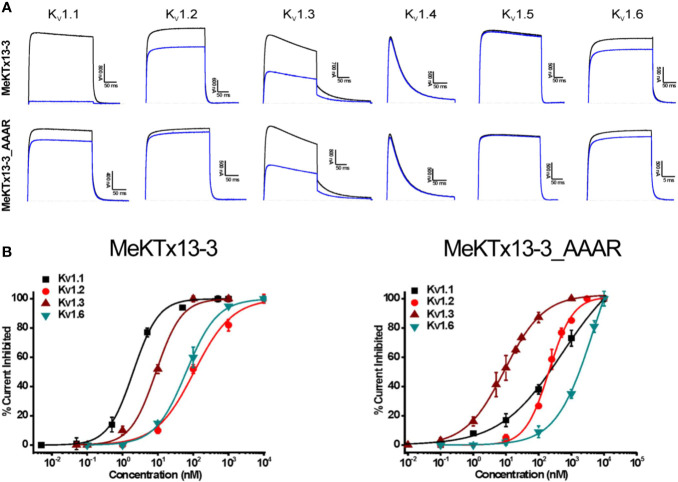
Electrophysiological profiling of MeKTx13-3 and MeKTx13-3_AAAR activities. **(A)** Representative traces of currents through KV1.1–1.6 in control (black) and after application of 10 nM toxin (blue). **(B)** Concentration–response curves of MeKTx13-3 (left) and MeKTx13-3_AAAR (right) on KV1.1–1.3 and 1.6 obtained by electrophysiological measurements. IC50 values are listed in **Table 1**.

We then estimated the activities of the obtained recombinant peptides MeKTx13-3 and MeKTx13-3_AAAR at a concentration of 10 nM against six isoforms of K_V_s (K_V_1.1–1.6) expressed in *X. laevis* oocytes (**Figure 3A**). Recombinant MeKTx13-3 as well as the natural toxin inhibited almost completely (by >95%) potassium currents through K_V_1.1; ~25%, ~50%, and ~15% of current through K_V_1.2, 1.3, and 1.6 was blocked. At the same concentration of 10 nM MeKTx13-3_AAAR blocked K_V_1.3 by ~50%, whereas only ~15%, ~5% and ~10% was blocked in K_V_1.1, 1.2 and 1.6. Neither K_V_1.4 nor K_V_1.5 were affected by any of the peptides.

Finally, we constructed dose–response curves for the susceptible channels (**Figure 3B**; see **Table 1** for IC_50_ values and Hill coefficients). MeKTx13-3_AAAR demonstrated a comparable activity with native MeKTx13-3 on K_V_1.3 (IC_50_ = 8.9 ± 0.9 nM for the natural toxin and 9.1 ± 0.4 nM for the mutant), whereas its affinity to K_V_1.1 decreased dramatically (IC_50_ = 541.5 ± 48.6 nM instead of 1.9 ± 0.2 nM for natural MeKTx13-3). MeKTx13-3_AAAR also showed reduced activity on K_V_1.2 (IC_50_ = 208.2 ± 15.2 nM compared to 105.9 ± 14.6 nM for MeKTx13-3) and K_V_1.6 (IC_50_ = 1522.3 ± 183.4 nM instead of 63.4 ± 4.5 nM).

**Table 1:** IC_50_ Values (in nM) Calculated for MeKTx13-3 and Its Derivative against K_V_1.1–1.6.

Toxin	K_V_1.1	K_V_1.2	K_V_1.3	K_V_1.4	K_V_1.5	K_V_1.6
MeKTx13-3	1.9 ± 0.2 *(0.9 ± 0.1)*	105.9 ± 14.6 *(1.3 ± 0.3)*	8.9 ± 0.9 *(0.8 ± 0.2)*	N/A	N/A	63.4 ± 4.5 *(1.0 ± 0.1)*
MeKTx13-3_AAAR	541.5 ± 48.6 *(0.7 ± 0.1)*	208.2 ± 15.2 *(1.2 ± 0.1)*	9.1 ± 0.4 *(0.7 ± 0.2)*	N/A	N/A	1522.3 ± 183.4 *(1.4 ± 0.3)*

N/A means toxin was not active up to 1 µM concentration. Hill coefficient values are given in parenthesis.

## Discussion

Animal venom serves a rich source of promising compounds affecting ion channels, which can be utilized as potential drug hits (Wulff et al., 2019). Detailed studies based on either toxin or channel mutagenesis are essential for (i) the understanding of fine molecular contacts between the toxins and very close channel isoforms, and (ii) design and production of more selective ligands. Hence prediction of critical amino acids involved in toxin–channel complex formation *in silico* is a convenient and powerful approach for following mutagenesis studies (Kuyucak and Norton, 2014). For instance, such computationally guided assay helped to design highly selective peptide drug hits or leads, such as ShK-192 (Pennington et al., 2009) and HsTX1[R14A] (Rashid et al., 2015).

Here, we have designed and produced a derivative of scorpion toxin MeKTx13-3 with its selectivity switched from K_V_1.1 to K_V_1.3. MeKTx13-3 is one of a limited number of known animal toxins that possess selectivity to homotetrameric K_V_1.1 (Kuzmenkov et al., 2019). We introduced several substitutions according to computer modeling experiments. To convert the selectivity of MeKTx13-3 we replaced three amino acids by alanine (Gln12Ala, Lys15Ala, and Lys18Ala) to abolish the formation of H-bonds, salt bridges, or cation-π interactions in the complexes with K_V_1.1 and 1.2. Moreover, to prevent the electrostatic repulsion between the negatively charged Asp33 in MeKTx13-3 and the conserved aspartic acid residue in the P-S6 loop of K_V_s we introduced the Asp33Arg replacement.

We produced not only MeKTx13-3_AAAR, but also recombinant MeKTx13-3 to confirm that (i) the peptide folding is correct, and (ii) lack of the C-terminal amidation does not impact toxin activity dramatically. These points are of importance, because misfolded toxins usually lose activity, whereas C-terminal amidation can boost ligand potency (Lebrun et al., 1997; Coelho et al., 2014). Voltage-clamp recordings in *X. laevis* oocytes showed that recombinant MeKTx13-3 is ~3.5 times less potent than the natural toxin. We attribute this decrease in activity to the amidation. It is well known that this post-translational modification can affect the activity of peptides. The effects may vary from dramatic to negligible, with most apparent cases found in hormones (Merkler, 1994). As for potassium channel blockers, the C-terminal amidation of ShK from the sea anemone *Stichodactyla helianthus* resulted in ~4 times decreased potency against K_V_1.3 (Pennington et al., 2012). Conversely, the amidation of HsTX1 (α-KTx 6.3) from the scorpion *Heterometrus spinifer* increases the activity against K_V_1.3 five-fold (Lebrun et al., 1997). The effects in our case are subtle, and we decided to use the recombinantly produced peptide MeKTx13-3 lacking the amidation in further studies.

The designed derivative MeKTx13-3_AAAR was also tested on six K_V_ isoforms and as we expected the selectivity of this analogue shifted towards K_V_1.3. A graphic approach to estimate toxin specificity to a particular channel isoform (K_V_1.3 in our case) is using the selectivity factor, i.e. the ratio of IC_50_ (or K_d_) values for two channels. MeKTx13-3 displays K_V_1.1/K_V_1.3 selectivity factor of approximately 0.2, while for MeKTx13-3_AAAR this parameter has changed to 60 (**Supplemental Figure S4**). There is a number of more K_V_1.3-specific toxins and their derivatives, for instance, HsTX1, Vm24, or moka1, demonstrating selectivity factors (K_V_1.1/K_V_1.3) of 500 or even 1000 (Romi-Lebrun et al., 1997; Takacs et al., 2009; Varga et al., 2012).

We aligned the sequences of several dozen toxins from KTx subfamilies α-KTx1–4, 11, 12, 15–18, and 21–24 with comparable length and cysteine pattern to MeKTx13-3 and found that two of the residues mutated in our study (Gln12, the first amino acid before the second Cys residue; and Lys18, the first amino acid after the third Cys) are quite conserved. In numerous toxins Gln/Glu and Lys/Arg are located in these positions, respectively. On the other hand, Lys15 between second and third Cys and Asp33 between the fifth and sixth Cys are variable. Within α-KTx 3 subfamily (to which MeKTx13-3 belongs) these positions display a similar pattern (see **Supplemental Figure S4**). All four residues seem to contribute to the bioactive surface of the toxins, and at present, there is no apparent explanation as to why two of them are more conserved than the other two. Moreover, we cannot infer any correlation between these residues and toxin potency or selectivity.

One may argue that the same substitutions as we introduced to MeKTx13-3 might bring about similar changes of selectivity in other α-KTx 3 toxins. However, and quite unfortunately, our current understanding of K_V_–KTx interactions does not allow to predict the specificity of toxins from primary structure. It appears that in each case molecular modeling experiments and a detailed analysis of the contacts are required. This is because one change in the sequence may actually affect how other residues interact—due to sterical hindrances or packing effects, electrostatic attraction or repulsion, H-bond reshuffling, or local folding rearrangements, all of which are not easily discernible from primary structures. For instance, in case of MeKTx13-3 Lys15 seems to make a salt bridge with Glu353 in K_V_1.1 (**Figure 1B**). Simple consideration of the primary structures would predict the same salt bridge in K_V_1.2 since it has Asp351 in the same position of the alignment (**Figure 1D**). This contact is not established in molecular modeling however, due to the neighboring Arg350, which repulses Lys15. Similarly, MeKTx13-3 residues Lys18 and Gln12 make channel-specific contacts due to the different folding of the channel extracellular loops, not just the amino acid substitutions. In conclusion, we hope to have demonstrated here the possibility of switching toxin specificity between two very close channel isoforms based on careful *in silico* design.

## Data Availability Statement

The datasets generated for this study can be found in the article/**Supplementary Material**.

## Ethics Statement

This study strictly complied with the World Health Organization’s International Guiding Principles for Biomedical Research Involving Animals. The research was carried out in AAALAC accredited organization according to the standards of the Guidefor Care and Use of Laboratory Animals (8th edition, Institute forLaboratory Research of Animals). The use of the frogs was in accordance with the license number LA1210239 of the Laboratory of Toxicology & Pharmacology, University of Leuven. The use of Xenopus laevis was approved by the Ethical Committee for animal experiments of the University of Leuven (P186/2019). All animal care and experimental procedures agreed with the guidelines of ‘European convention for the protection of vertebrate animals used for experimental and other scientific purposes’ (Strasbourg, 18.III.1986).

## Author Contributions

AK and AV designed research. AG, AK, EP-J, SP, VT, and AV analyzed data. AJK, EP-J, SP, VT, AG, and AC performed research. AK, VT, and AV wrote the paper. RE and JT supervised molecular modeling and electrophysiology, respectively.

## Funding

This work was supported by the Russian Science Foundation (grant no. 18-74-00125). JT was funded by grants G0E7120N, GOC2319N and GOA4919N from the F.W.O Vlaanderen. SP was supported by KU Leuven funding (PDM/19/164). Fundação de Amparo à Pesquisa do Estado de São Paulo (São Paulo Research Foundation, Brazil, scholarship to EP-J n. 2016/04761-4), and Coordenação de Aperfeiçoamento de Pessoal de Nível Superior (Coordination for the Improvement of Higher Education Personnel, Brazil; scholarship to EP-J n. 88881.186830/2018-01).

## Conflict of Interest

The authors declare that the research was conducted in the absence of any commercial or financial relationships that could be construed as a potential conflict of interest.
