# Supplementary material for: Tuning Scorpion Toxin Selectivity: Switching From KV1.1 to KV1.3
Source: Front Pharmacol. 2020 Jul 7;11:1010. doi: 10.3389/fphar.2020.01010 (PMC7358528; doi:10.3389/fphar.2020.01010)
Supplement: Supplementary file 1 [file DataSheet_1.docx]

**MeKTx13-3**

Kpn1 D D D D R V G I N V K **C** K H S G Q **C** L K P **C** K D A G M R F G K **C** I N G K **C** D **C** T P K end BamHI

GCGATAGGTACCGACGATGACGATCGTGTGGGCATTAATGTGAAATGC CAGTGCCTGAAACCGTGCAAAGATGCGGGCATGCGTTTTGGCAAATGC

GCGATAGGTACCGACGATGACGATCGTGTGGGCATTAATGTGAAATGCAAACATTCCGGCCAGTGCCTGAAACCGTGCAAAGATGCGGGCATGCGTTTTGGCAAATGCATTAATGGCAAATGCGATTGCACCCCGAAATAGGGATCCGCGATA

CGCTATCCATGGCTGCTACTGCTAGCACACCCGTAATTACACTTTACGTTTGTAAGGCCGGTCACGGACTTTGGCACGTTTCTACGCCCGTACGCAAAACCGTTTACGTAATTACCGTTTACGCTAACGTGGGGCTTTATCCCTAGGCGCTAT

CCCGTAATTACACTTTACGTTTGTAAGGCCGGTCACGGACTTTGGCACGT CGCAAAACCGTTTACGTAATTACCGTTTACGCTAACGTGGGGCTTTATCCCTAGGCGCTAT

**MeKTx13-3_AAAR**

Kpn1 D D D D R V G I N V K **C** K H S G A **C** L A P **C** A D A G M R F G K **C** I N G K **C** R **C** T P K end BamHI

GCGATAGGTACCGACGATGACGATCGTGTGGGCATTAATGTGAAATGC TGTCTGGCACCGTGTGCAGATGCGGGCATGCGTTTTGGCAAATGCATT

GCGATAGGTACCGACGATGACGATCGTGTGGGCATTAATGTGAAATGCAAACATTCCGGCGCGTGTCTGGCACCGTGTGCAGATGCGGGCATGCGTTTTGGCAAATGCATTAATGGCAAATGCCGTTGCACCCCGAAATAGGGATCCGCGATA

CGCTATCCATGGCTGCTACTGCTAGCACACCCGTAATTACACTTTACGTTTGTAAGGCCGCGCACAGACCGTGGCACACGTCTACGCCCGTACGCAAAACCGTTTACGTAATTACCGTTTACGGCAACGTGGGGCTTTATCCCTAGGCGCTAT

CCCGTAATTACACTTTACGTTTGTAAGGCCGCGCACAGACCGTGGCACAC CGCAAAACCGTTTACGTAATTACCGTTTACGGCAACGTGGGGCTTTATCCCTAGGCGCTAT

**Figure S1.** Construction of DNA fragments encoding recombinant MeKTx13-3 and its mutant MeKTx13-3_AAAR from oligonucleotides. Restriction sites are shown in blue, stop codons are in red, enteropeptidase cleavage site-encoding codons are in green, and the mutated codons are shown in yellow. Synthetic oligonucleotides used for PCR are shown above and below the full sequences (see also **Table S1**).


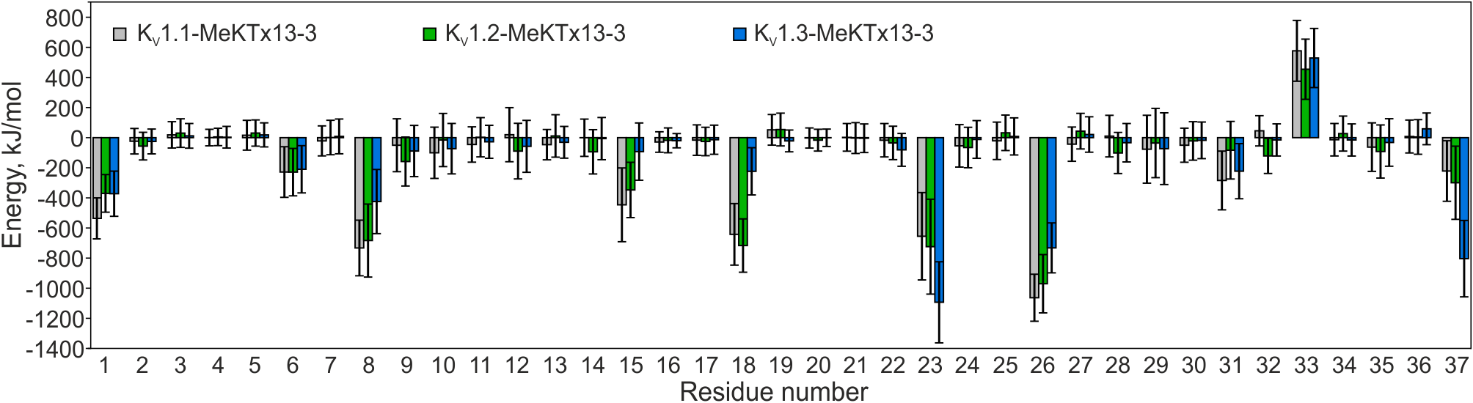


**Figure S2.** Interaction energy profiles of MeKTx13-3 in complex with K_V_1.1–1.3. Bar charts show residual contributions to the interaction energy averaged over MD simulation. Error bars indicate standard deviations.


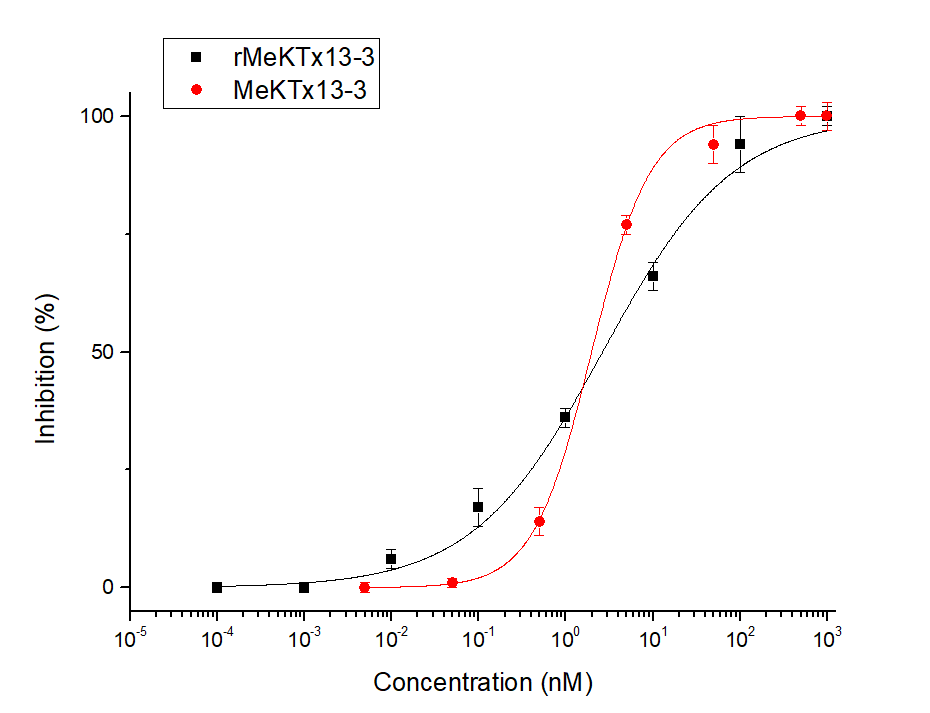


**Figure S3.** Concentration–response curves of recombinant (black squares) and natural (red circles) MeKTx13-3 on K_V_1.1 obtained by electrophysiological measurements. IC_50_ values are listed in **Table S3**.


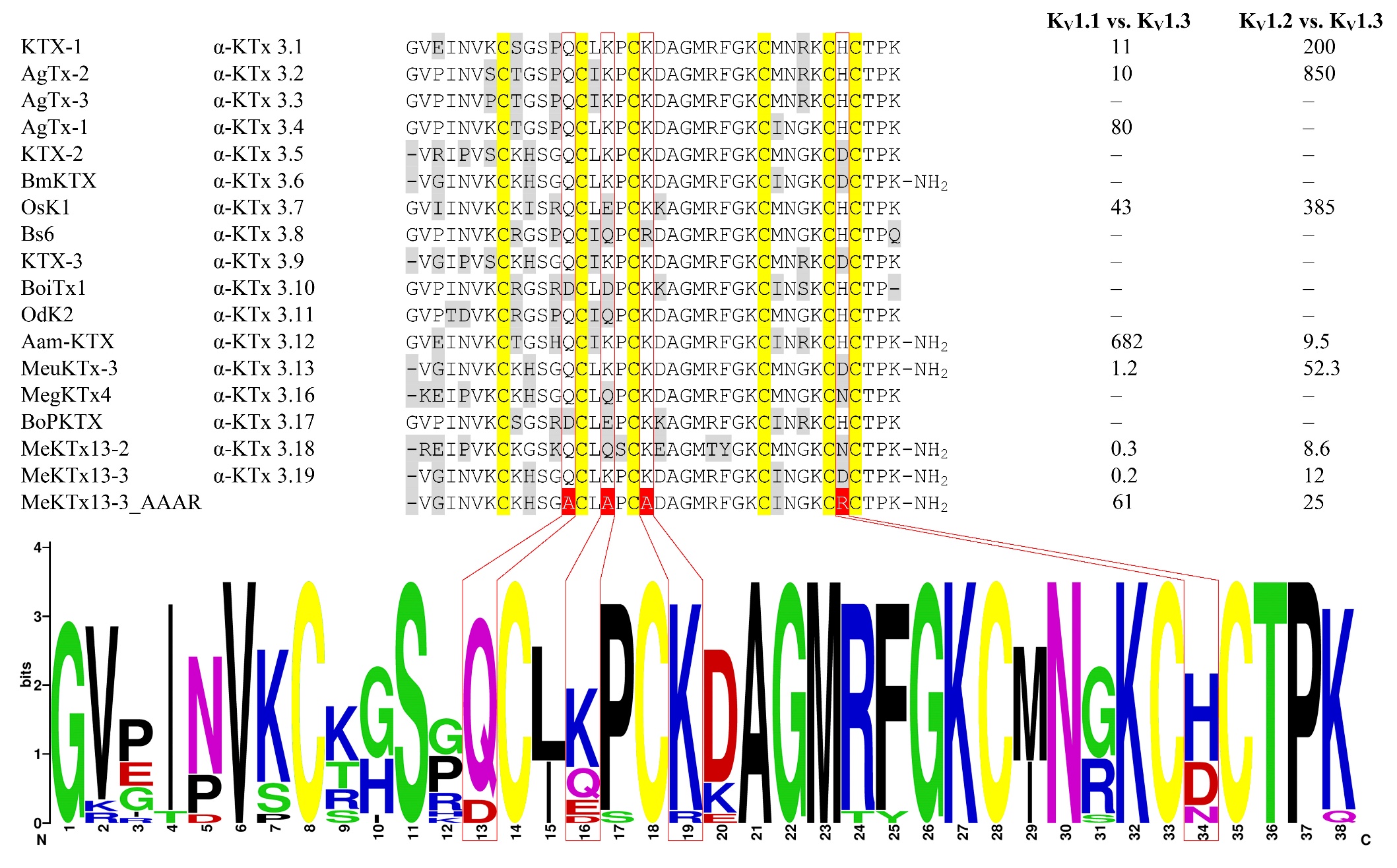


**Figure S4.** Amino acid sequence alignment of toxins from α-KTx 3 group and artificial derivative MeKTx13-3_AAAR. Different residues are shaded gray, and cysteine residues are shown on a yellow background. Red frames indicate positions where mutations were introduced. On the bottom a graphical representation of the multiple sequence alignment is presented. The sequence logo was built by WebLogo 3 (<https://weblogo.berkeley.edu/>) using the algorithm developed by Schneider and Stephen (Schneider et al., 1990). On the right selectivity factors (ratios between IC_50_ values for two channels) for both pairs K_V_1.1 vs. K_V_1.3 and K_V_1.2 vs. K_V_1.3 are listed.

**Table S1.** Oligonucleotides that were used to amplify DNA fragments encoding recombinant MeKTx13-3 and its mutant MeKTx13-3_AAAR (see **Figure S1**). Restriction sites are shown in blue, stop codons are in red, enteropeptidase cleavage site-encoding codons are in green, and the mutated codons are shown in yellow.

| **Name** | **Nucleotide sequence 5’-3’** |
| --- | --- |
| F1-1 | GCGATAGGTACCGACGATGACGATCGTGTGGGCATTAATGTGAAATGC |
| F2-1 | CAGTGCCTGAAACCGTGCAAAGATGCGGGCATGCGTTTTGGCAAATGC |
| F2-2 | TGTCTGGCACCGTGCGCAGATGCGGGCATGCGTTTTGGCAAATGCATT |
| R1-1 | TATCGCGGATCCCTATTTCGGGGTGCAATCGCATTTGCCATTAATGCATTTGCCAAAACGC |
| R1-2 | TATCGCGGATCCCTATTTCGGGGTGCAACGGCATTTGCCATTAATGCATTTGCCAAAACGC |
| R2-1 | TGCACGGTTTCAGGCACTGGCCGGAATGTTTGCATTTCACATTAATGCCC |
| R2-2 | CACACGGTGCCAGACACGCGCCGGAATGTTTGCATTTCACATTAATGCCC |

**Table S2.** Specific interactions (H-bonds, salt bridges, stacking/π-π, and cation-π) observed in MD of MeKTx13-3 complexes with K_V_1.1–1.3 (500 ns). All contacts in this table are “aligned” according to the amino acid sequence alignment of the channels. Each contact in each complex is put in correspondence to analogous contacts in other complexes. In case when analogous contacts are not observed during MD, the corresponding cell in the table is empty. Lifetime of each contact was estimated as part of the full MD trajectory time (300 ns in total, starting after 200 ns of MD: first 200 ns were not taken into account to get representative data); “s” stands for short-living contacts: lifetime is less than 10%; “m”, medium-living contacts, lifetime is less than 50%; “l”, long-living contacts, lifetime is greater than 50%. Subunit identifiers (A, B, C, or D) are merged with the name and number of channel residues (e.g. Glu349C). Long- and medium-living contacts observed only in complexes of MeKTx13-3 with K_V_1.1 and 1.2 are shown on a blue background.

| **Hydrogen bonds** | | | | | | | | |
| --- | --- | --- | --- | --- | --- | --- | --- | --- |
| **K_V_1.1–MeKTx13-3** | | | **K_V_1.2–MeKTx13-3** | | | **K_V_1.3–MeKTx13-3** | | |
| Peptide residue | Channel residue | Lifetime | Peptide residue | Channel residue | Lifetime | Peptide residue | Channel residue | Lifetime |
|  |  |  | Lys8 | Glu349C | l |  |  |  |
| Lys8 | Ala352C | m |  |  |  |  |  |  |
| Lys8 | Glu353C | m | Lys8 | Asp351C | m |  |  |  |
| Lys8 | Ser354C | m |  |  |  |  |  |  |
| Lys8 | His355C | s |  |  |  |  |  |  |
|  |  |  |  |  |  | Lys8 | Asp383B | s |
|  |  |  |  |  |  | Lys8 | Asp399B | l |
| Lys8 | Tyr379C | m |  |  |  |  |  |  |
|  |  |  | Lys8 | Thr379C | s |  |  |  |
|  |  |  | His9 | Asp351C | l |  |  |  |
|  |  |  |  |  |  | His9 | Ser376C | m |
|  |  |  |  |  |  | His9 | His401C | s |
|  |  |  | Ser10 | Gln353C | l |  |  |  |
|  |  |  | Ser10 | Gly374C | m | Ser10 | Gly398C | l |
| Ser10 | Asp377C | l | Ser10 | Asp375C | l | Ser10 | Asp399C | m |
|  |  |  |  |  |  | Ser10 | His401C | m |
|  |  |  | Gly11 | Gln353C | s |  |  |  |
|  |  |  | Gln12 | Asp351C | m |  |  |  |
| Lys15 | Glu353A | m |  |  |  |  |  |  |
|  |  |  |  |  |  |  |  |  |
| Lys18 | Ser354A | m |  |  |  |  |  |  |
|  |  |  | Lys18 | Gln353A | m |  |  |  |
|  |  |  |  |  |  | Arg23 | Glu370A | l |
|  |  |  |  |  |  | Arg23 | Asp372A | l |
|  |  |  |  |  |  | Arg23 | Asp373A | m |
|  |  |  | Arg23 | Gln353A | s |  |  |  |
| Arg23 | Asp377A | l | Arg23 | Asp375A | l |  |  |  |
| Lys26 | Tyr375A | l | Lys26 | Tyr373A | m |  |  |  |
| Lys26 | Tyr375B | l | Lys26 | Tyr373C | m | Lys26 | Tyr397C | m |
| Lys26 | Tyr375C | m | Lys26 | Tyr373D | l |  |  |  |
| Lys26 | Tyr375D | s |  |  |  |  |  |  |
| Cys27 | Tyr379C | m |  |  |  |  |  |  |
|  |  |  | Asn29 | Gln353B | l |  |  |  |
|  |  |  |  |  |  | Asn29 | Asp399D | m |
| Asn29 | Gly376B | m |  |  |  |  |  |  |
| Asn29 | Asp377B | l | Asn29 | Asp375B | m | Asn29 | Asp399B | m |
|  |  |  | Asn29 | Val377B | m |  |  |  |
|  |  |  | Thr35 | Gly374A | l |  |  |  |
| Thr35 | Asp377A | m |  |  |  | Thr35 | Asp399A | l |
|  |  |  |  |  |  | Lys37 | Phe378A | s |
|  |  |  |  |  |  | Lys37 | Asp383A | l |
|  |  |  |  |  |  | Lys37 | Asp399A | l |
|  |  |  |  |  |  | Lys37 | His401A | l |
|  |  |  |  |  |  |  |  |  |
| **Salt bridges** | | | | | | | | |
| **K_V_1.1–MeKTx13-3** | | | **K_V_1.2–MeKTx13-3** | | | **K_V_1.3–MeKTx13-3** | | |
| Peptide residue | Channel residue | Lifetime | Peptide residue | Channel residue | Lifetime | Peptide residue | Channel residue | Lifetime |
|  |  |  | Lys8 | Glu349C | m |  |  |  |
| Lys8 | Glu353C | m | Lys8 | Asp351C | m |  |  |  |
|  |  |  |  |  |  | Lys8 | Asp383B | s |
|  |  |  |  |  |  | Lys8 | Asp399B | l |
| Lys15 | Glu353A | m |  |  |  |  |  |  |
|  |  |  |  |  |  | Arg23 | Glu370A | l |
|  |  |  |  |  |  | Arg23 | Asp372A | l |
|  |  |  |  |  |  | Arg23 | Asp373A | m |
| Arg23 | Asp361A | m |  |  |  |  |  |  |
| Arg23 | Asp377A | l | Arg23 | Asp375A | l |  |  |  |
|  |  |  |  |  |  | Lys37 | Asp383A | l |
|  |  |  |  |  |  | Lys37 | Asp399A | m |
|  |  |  |  |  |  |  |  |  |
| **Stacking / π-π interactions** | | | | | | | | |
| **K_V_1.1–MeKTx13-3** | | | **K_V_1.2–MeKTx13-3** | | | **K_V_1.3–MeKTx13-3** | | |
| Peptide residue | Channel residue | Lifetime | Peptide residue | Channel residue | Lifetime | Peptide residue | Channel residue | Lifetime |
| His9 | His355C | m |  |  |  |  |  |  |
|  |  |  |  |  |  | His9 | His401C | m |
|  |  |  |  |  |  | Arg23 | Phe378A | l |
| Arg23 | Tyr379D | s |  |  |  |  |  |  |
| Phe24 | His355A | m |  |  |  |  |  |  |
| Phe24 | Tyr379A | m |  |  |  | Phe24 | His401A | l |
|  |  |  |  |  |  |  |  |  |
| **Cation-π interactions** | | | | | | | | |
| **K_V_1.1–MeKTx13-3** | | | **K_V_1.2–MeKTx13-3** | | | **K_V_1.3–MeKTx13-3** | | |
| Peptide residue | Channel residue | Lifetime | Peptide residue | Channel residue | Lifetime | Peptide residue | Channel residue | Lifetime |
| Lys8 | His355C | m |  |  |  |  |  |  |
| Lys8 | Tyr379C | m |  |  |  |  |  |  |
| Lys18 | His355A | m |  |  |  |  |  |  |
| Arg23 | His355A | s |  |  |  |  |  |  |
|  |  |  |  |  |  | Arg23 | Phe378A | l |
| Arg23 | Trp364A | m |  |  |  |  |  |  |
| Arg23 | Tyr379D | l |  |  |  |  |  |  |
| Lys26 | Tyr379A | m |  |  |  | Lys26 | His401A | m |
|  |  |  |  |  |  |  |  |  |

**Table S3.** Activity comparison of natural and recombinant MeKTx13-3 on K_V_1.1. IC_50_ values in nM and Hill coefficients are indicated.

| **Toxin** | **K_V_1.1** | **Hill** |
| --- | --- | --- |
| Natural MeKTx13-3 | 1.9 ± 0.2 | 0.9 ± 0.1 |
| Recombinant MeKTx13-3 | 6.7 ± 2.7 | 0.6 ± 0.1 |
